# *Lycium barbarum* polysaccharides in ageing and its potential use for prevention and treatment of osteoarthritis: a systematic review

**DOI:** 10.1186/s12906-021-03385-0

**Published:** 2021-08-17

**Authors:** Junguo Ni, Manting Au, Hangkin Kong, Xinluan Wang, Chunyi Wen

**Affiliations:** 1 Department of Biomedical Engineering, Faculty of Engineering, Hong Kong Polytechnic University, Kowloon, Hong Kong; 2 Department of Applied Biology and Chemical Technology, Hong Kong Polytechnic University, Kowloon, Hong Kong; 3 Centre for Translational Medical Research and Development, Shenzhen Institutes of Advanced Technology, Chinese Academy of Science, Shen Zhen, China

**Keywords:** *Lycium Barbarum* polysaccharides, Reactive oxidative species, Mitochondrial dysfunction, Cellular senescence, Osteoarthritis

## Abstract

**Background:**

*Lycium barbarum* polysaccharide (LBP), the most abundant functional component of wolfberry, is considered a potent antioxidant and an anti-ageing substance. This review aims to outline the hallmarks of ageing in the pathogenesis of osteoarthritis (OA), followed by the current understanding of the senolytic effect of LBP and its potential use in the prevention and treatment of OA. This will be discussed through the lens of molecular biology and herbal medicine.

**Methods:**

A literature search was performed from inception to March 2020 using following keywords: “*Lycium barbarum* polysaccharide”, “DNA damage”, antioxidant, anti-apoptosis, anti-inflammation, anti-ageing, osteoarthritis, chondrocytes, fibroblasts, osteoblasts, osteoclasts, and “bone mesenchymal stem cell”. The initial search yielded 2287 papers, from which 35 studies were selected for final analysis after screening for topic relevancy by the authors.

**Results:**

In literature different in vitro and in vivo ageing models are used to demonstrate LBP’s ability to reduce oxidative stress, restore mitochondrial function, mitigate DNA damage, and prevent cellular senescence. All the evidence hints that LBP theoretically attenuates senescent cell accumulation and suppresses the senescence-associated secretory phenotype as observed by the reduction in pro-inflammatory cytokines, like interleukin-1beta, and matrix-degrading enzymes, such as MMP-1 and MMP-13. However, there remains a lack of evidence on the disease-modifying effect of LBP in OA, although its chondroprotective, osteoprotective and anti-inflammatory effects were reported.

**Conclusion:**

Our findings strongly support further investigations into the senolytic effect of LBP in the context of age-related OA.

## Background

Osteoarthritis (OA) is one of the fastest growing disabilities worldwide and is typically associated with irregular chronic pain which affects patients’ quality of life. OA has attracted many scientists throughout the centuries to explore its underlying mechanisms. However, to date there is still no disease-modifying drug available [1]. Consequently, identifying risk factors and selecting the right targets remain a great and unmet challenge in the OA field [2]. Today, OA research has confirmed that among others, hypertension, obesity and joint injury are associated with the induction of OA [2–5].

Evidence showed that the prevalence of OA increases with age. Different studies found that the incidence of OA dramatically increased with age both in women and men from all regions of the world [6, 7]. It affects an estimated 10% of men and 18% of women over 60 years old [6]. At present, with the rapid ageing population, the global incidence of OA is also rising steadily and it is estimated that 67 million people in the United States will be affected by 2030 [8]. All the evidence confirms OA as an age-related disease.

Hence, over the past decade, the role of ageing in the pathogenesis of OA has been intensively investigated [9, 10]. According to Lopez-Otin et al., there are nine hallmarks of ageing: genomic instability, telomere attrition, epigenetic alterations, loss of proteostasis, deregulated nutrient-sensing, mitochondrial dysfunction, cellular senescence, stem cell exhaustion, and altered intercellular communication. Most of them are caused by oxidative stress and reactive oxygen species (ROS) whose imbalance is a key feature of ageing [11]. In the pathogenesis of OA, most of these hallmarks, including genomic instability, mitochondrial dysfunction, and cellular senescence, have been studied [12]. For example, rapid increase of ROS leads to mitochondrial DNA (mtDNA) damage and mitochondrial dysfunction, causing premature chondrocyte senescence and apoptosis, which eventually increases the risk of OA [13–15]. Therefore, blocking major senescence signalling pathways in OA pathogenesis could be a valid method in delaying the onset of OA meanwhile it also creates the possibility of using anti-ageing reagents as emerging drug candidates for OA treatment.

*Lycium barbarum* (LB, Gouqi, wolfberry, or Fructus lycii) is a well-known traditional herb with widespread distribution [16]. It is extremely important in China and other Asian countries, not only because it can be used as nutritional supplement in daily life, but also because of its medicinal value [17–19]. Especially in recent years, with the in-depth study of LB, it has been highly valued by Chinese and foreign medical scientists and dietetic health experts for its antioxidant and anti-ageing effects [20–26].

*Lycium barbarum* polysaccharide (LBP), the most abundant functional component of wolfberry [27], is an important functional additive of dietary supplements and plays an important role in the anti-ageing and antioxidant function of LB. Xue S, et al. reported that a dose of 220 μg/mL and a dose of 440 μg/mL LBP could reverse the H_2_O_2_-induced oxidative injury [28]. Another study suggested that LBP could extend the average life span of *Drosophila melanogaster* due to an increase in antioxidant activity, i.e. an upregulation of the SOD and CAT levels [29]. Moreover, LBP has been studied in the reproductive system [30–37] as well as in the cells and tissues derived from different germ layers [38–48] to evaluate its protective value. Again, LBP performed excellently in scavenging free radicals, maintaining mitochondrial function and showed exquisite antioxidant effects. Recently, numerous mice models also showed the same results [49–53]. Overall, these findings demonstrated that LBP has good ROS-reducing and antioxidant properties in different in vivo and in vitro models. This will restore the oxidative stress balance and finally attenuate ageing to some extent. Based on its properties, we hypothesized that LBP could act as an anti-ageing reagent that may have the potential to prevent age-related OA.

Although the pathological mechanism of ageing-associated OA and LBP anti-ageing effects have been discussed for several years, the direct effects of LBP in the treatment of OA are relatively less studied up to this moment [54–57]. Therefore, in this review, we provided an overview of the pathogenesis of OA focussing on the role of genomic instability, mitochondrial dysfunction and cellular senescence. Next, we systematically searched the published studies for the LBP anti-ageing effects in different models. Additionally, we attempted to postulate the beneficial effect of LBP on the pathogenesis of OA. Finally, we proposed LBP as a potential treatment against OA and try to provide a new horizon for pharmaceutical scientists.

## Methods

**Fig. 1:**
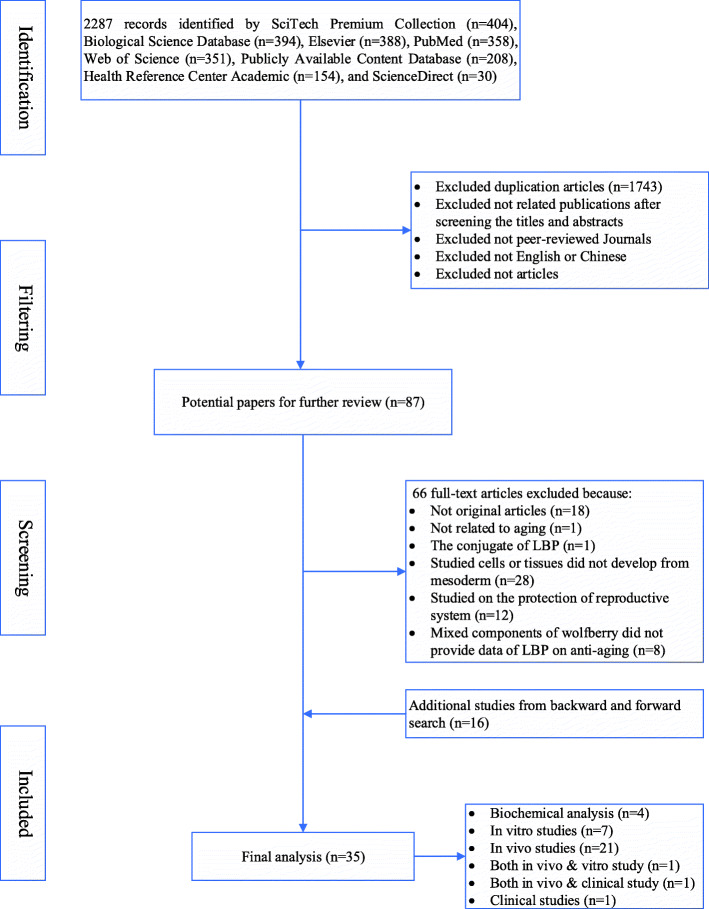
The flowchart of literature search and systemic review

The workflow of our systematic review on LBP has been illustrated in Fig. 1. In brief, the search was performed in March 2020 by using the keyword “*Lycium barbarum* polysaccharide” combined with “DNA damage”, antioxidant, anti-apoptosis, anti-inflammation, anti-ageing, osteoarthritis, chondrocytes, fibroblasts, osteoblasts, osteoclasts, and “Bone Mesenchymal Stem Cell”. A total of 2287 records were identified by different databases. The filter was set to select all articles published in peer-reviewed journals, which were available in full text and written in English or Chinese, excluding duplication articles, resulting in 544 articles. Next, a screening for topic relevance through title and article abstracts was performed. In the end, 35 studies were selected for final analysis, including in vitro studies, animal studies, and clinical studies.

## Results

### Hallmarks of ageing in the pathogenesis of OA

OA is a degenerative joint disease characterized by cartilage degeneration, synovial hypertrophy and functional ligament damage. The occurrence of OA is a complex biological process in which multiple factors, such as genetic factors, gender, diet, obesity and age, play a role [58]. Among them, advanced age is most closely related to OA [2]. In-depth studies showed that oxidative stress and excessive accumulation of ROS are important factors leading to ageing and that they are involved in almost all hallmarks of the ageing process [11, 59, 60]. In the following, different hallmarks of ageing, including genomic instability (DNA damage and telomere attrition), mitochondrial dysfunction, cellular senescence, and other factors involved in cartilage degradation in the pathogenesis of OA will be discussed.

#### Genomic instability (telomere attrition and DNA damage)

Genomic instability is the most direct hallmark of ageing in OA. In the classic replicative senescence hypothesis, telomere attrition is one of the major markers of ageing. As the cell undergoes mitosis, the telomere length gradually shortens until it reaches the minimum length required for replication, eventually leading to cell cycle stagnation [61]. As early as the beginning of the twenty-first century, telomere erosion has been confirmed in isolated human cartilage, and it has become more serious with age [7]. The research findings supported that there was a partial association between OA and replicative senescence [7]. However, not all elderly suffers from OA, which means that in addition to telomere erosion caused by replicative senescence, there are other forms of telomere damage, which may be the main cause of the onset of OA [62–65].

Fortunately, more and more researchers have paid attention to this problem in recent years, and a large amount of evidence has emerged to prove that exogenously induced cellular senescence, also known as stress-induced senescence, is associated with OA [59, 65–68]. There is evidence showing that a high concentration of oxygen leads to premature senescence of human articular chondrocytes through increased telomere erosion and mtDNA damage [66]. Telomere shortening or DNA damage activates tumour protein p53 which later promotes the expression of p21 and p16, ultimately leading to cellular senescence [3]. Subsequently, another experiment explained that ROS accelerates the senescence of human chondrocytes by inducing telomere instability which is responsible for the occurrence of OA [65]. Of note, the level of ROS is a critical factor in ageing. The sensitivity of telomere terminus to ROS plays an essential role in the pathogenesis of OA.

#### Mitochondrial dysfunction

Mitochondrial dysfunction, another manifestation of ageing, occurs in response to damage, including oxidative and inflammatory damage. It is also one of the important hallmarks of age-related OA.

The major cause of chondrocytes mitochondrial dysfunction is the rapid increase of ROS generation [69–72]. In OA chondrocytes, it has been observed that boosting ROS changed the adenosine triphosphate (ATP) synthesis and mitochondrial respiratory chain (MRC) activity, which ultimately lead to mitochondrial dysfunction [72]. Recently, it has been reported that mitochondria will rapidly release ROS and superoxide radicals after a single, blunt-impact injury performed to osteochondral explants in vitro, causing acute chondrocytes death and exacerbating OA characterization [70]. It is suggested that mitochondrial dysfunction is highly related to OA. A rabbit model demonstrated that mitochondrial dysfunction caused by advanced oxidation production products (AOPPs) could lead to chondrocytes apoptosis and aggravate the osteoarthritic symptoms of rabbit cartilage [71].

In addition, high expression of inflammatory factors in chondrocytes was another reason for mitochondrial dysfunction. Recent studies showed that upregulation of IL-1β and TNF-α could induce overexpression of NO which damaged mtDNA and reduced mitochondrial transcription [3, 73]. It also revealed that pro-inflammatory factors contribute to the process between mitochondrial damage and apoptosis in chondrocytes [3, 73]. Furthermore, other studies have shown that in the absence of autophagy, mtDNA mutations and reduced mtDNA repair capacity could also lead to mitochondrial dysfunction in chondrocytes, exacerbating the risk of OA [74–76].

Mitochondrial dysfunction may induce several pathological processes of OA, including oxidative stress response, chondrocyte apoptosis as well as acute chondrocytes death [77]. Taken together, inhibiting or repairing mitochondrial dysfunction could be an emerging strategy for OA.

#### Cellular senescence

Cellular senescence is one of the nine recognized hallmarks of ageing. Normally, somatic cells have a Hayflick limit, that is a maximum of ~ 50 divisions. This is followed by growth arrest which is called senescence [78]. The senescent cells (SnCs) remain viable and metabolically active but secrete a variety of pro-inflammatory cytokines, growth factors, chemokines and proteinases known as the senescence-messaging secretome or senescence-associated secretory phenotype (SASP) [79, 80]. Senescence is a protective mechanism in various physiological processes, for example it evokes tumour suppression and limits fibrosis in wound healing [81]. On the other hand, senescence of stem or progenitor cells will impair tissue regeneration, and SASP can damage the surrounding tissue [79]. Over the past decade, senescence has been linked with ageing and age-related pathologies [82, 83], including OA [12, 68].

The number of senescent cells increases gradually with age. Changes in signalling pathways associated with ageing in chondrocytes, the main cell type found in articular cartilage, will raise the risk of OA [7]. To maintain the normal function of joints, chondrocytes will continuously synthesize new matrix molecules throughout their lives. In addition, insulin-like growth factor-1 (IGF-1) and transforming growth factor-β (TGF-β) are indispensable signalling molecules in the synthesis and catabolism of articular cartilage. However, the anabolic response of rat chondrocytes to IGF-1 worsened with age, while the ability of chondrocytes to release IGF-1 binding protein increased [84]. It indicated that chondrocytes senescence would cause a dynamic imbalance in the synthesis and degradation of articular cartilage. Besides, chondrocytes senescence could also affect the TGF-β signalling pathway. An experiment showed that the expression of TGF-β family factor receptors bAlk2, bAlk3, bAlk4, bAlk5, and bBmpr2 decreased significantly with age in bovine articular cartilage, which eventually led to age-related cartilage thinning and collagen loss [85]. Moreover, chondrocyte anabolism is also affected by the NF-κB signalling pathway [86, 87]. Chondrocytes senescence resulted in the accumulation of advanced glycation end products (AGEs) [88], and the expression of AGEs could activate the NF-κB signalling pathway, increase the production of matrix metalloproteinase (MMP) -13, and finally cause degradation of articular cartilage [89]. Together, this demonstrated that changes in the chondrocyte signalling pathways will have a considerable impact on the incidence of OA.

The hallmark of OA is degeneration of articular cartilage and chondrocyte senescence is largely responsible for this phenomenon. In human OA cartilage lesions, SnCs were found near the cluster of chondrocytes [90], which exhibited characteristics of progenitor cells with increased proliferation [91, 92]. Adult articular chondrocytes have limited proliferation capacity. In response to altered mechanical loading [64, 93] or oxidative stress [65], articular chondrocytes underwent premature senescence with shortening of telomeres, which provoked the onset of OA [94]. It has been well documented that OA chondrocytes expressed a variety of the SnCs markers such as telomere attrition [7], activation of senescence-associated beta-galactosidase (SAβGal) [90], overexpression of p16^Ink4a^ [95] as well as MMP-1, 3 and 13 [96]. The percentage of SnCs cells in articular cartilage increased with the severity of knee OA [97]. Moreover, transplantation of SAβGal-positive SnCs into synovial joint led to an OA-like lesion in rodents [13]. Furthermore, ablation of p16^Ink4a^-positive SnCs using a genetically modified mice model could mitigate OA [94]. All the evidence suggests that the accumulation of SnCs impairs homeostasis of articular cartilage. Thus, removal of SnCs could be a promising therapeutic strategy for OA.

#### Other hallmarks of ageing in OA

Besides genomic instability, mitochondrial dysfunction and cellular senescence, inflammaging could be the other hallmark of ageing in OA. Inflammaging refers to chronic low-grade inflammation that develops with advanced age. Typically inflammaging is observed both locally and systemically in OA [98]. In addition, synovial inflammation and subchondral bone disturbance were often involved in the initiation of OA [99, 100]. Such inflammation will accelerate cellular senescence and make normal cells secrete SASP [101]. Recent studies have identified p16^Ink4a^-positive SnCs in inflamed synovium [94], cartilage surface [94] and aged bone microenvironment [102]. Besides, inflammatory mediators, like COX-2, IL-1β and TNF-α, were shown to be upregulated in the OA group when compared to the control group [103]. These results indicate that there is a strong association between inflammaging, senescence and OA.

In fact, inflammation plays a crucial part in OA development. Inflammaging in OA is caused by damage-associated molecular patterns (DAMPs), e.g. high-mobility group box 1 (HMGB1) protein, S100 family and uric acid [101, 104]. The DAMPs are mainly triggered by abnormal ROS accumulation [105]. The up-regulated DAMPs will then promote SASP factors release, i.e. MMPs and inflammatory cytokines, through mitogen-activated protein kinases (MAPK) and NF-κB signalling pathway [104, 105], which will finally provoke the OA progress. It is noteworthy that DAMPs can cause chronic low-grade inflammation in the joint and can accelerate cellular pro-senescence by cell-to-cell communication through NF-κB pathway [106].

Stem cell exhaustion was found to be another hallmark of age-related OA. The expression of p16^Ink4a^ was found to be upregulated in aged mesenchymal stem cells (MSCs) [107]. As bone marrow-derived mesenchymal stem cells (bmMSC) can differentiate into osteoblasts and chondrocytes for repair of aged cartilage, their functional senescence or depletion will contribute to the development of osteoarthritis [108]. An in vivo study has demonstrated that metformin-stimulated adipose tissue-derived human MSCs (Ad-hMSCs) could prevent the degeneration of cartilage effectively and prolong the survival time of MSCs in inflamed joints [109]. Articular cartilage stem cells (ACSCs) were known to repair articular cartilage [110]. However, with the development of OA, the population of ACSCs would gradually diminish [110]. As a whole, preventing stem cell exhaustion would be an effective way to prevent OA.

**Fig. 2:**
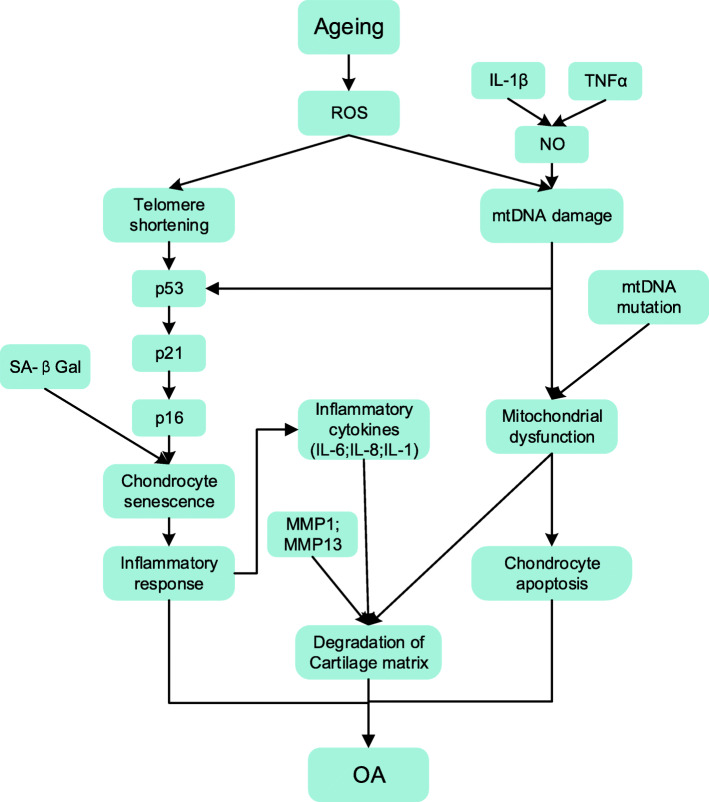
Schematic illustration of pathogenesis of OA in the literature. ROS (reactive oxygen species); NO (nitric oxide); mtDNA (mitochondrial deoxyribonucleic acid); TNF-α (tumor necrosis factor-α); IL-1β (interleukin-1β); IL-6 (interleukin-6); IL-8 (interleukin-8); IL-1 (interleukin-1); MMP-1 (matrix metalloproteinase-1); MMP-13 (matrix metalloproteinase-13); SA-βgal (Senescence-associated beta-galactosidase); OA (osteoarthritis)

*In short*, the pathogenesis of OA caused by ageing is characterized by oxidative damage: DNA damage, telomere attrition, mitochondrial dysfunction, cellular senescence, inflammaging, and stem cell exhaustion (Fig. 2). As age-related OA is a multifactorial disease, it is necessary to utilize therapy strategies which can target multiple signalling pathways to treat OA more efficiently.

### Evidence of LBP anti-ageing effects

*Lycium barbarum* (LB) is a traditional Chinese herbal medicine and has a complex chemical composition with extremely diverse targets. There are many bioactive substances in LB such as LBP, betaine, carotenoids, zeaxanthin, alkaloids, β-sitosterol, cerebroside, thiamine, riboflavin, flavonoids and phenolics [27, 111]. Among them, the polysaccharide content in LB (dried fruits) could reach as much as 5–8% [112]. LBP, as the most abundant one, has unique advantages: it is easy to extract, and its pharmacological characteristics are ideal for further research. So far, the most common isolation techniques for LBP from LB are through leaching extraction, microwave extraction, and ultrasonic extraction, by using water or ethanol as solvent [113]. Compared to the other active substances of LB, LBP is favoured because of its relatively simple extraction process. Moreover, it shows good anti-ageing properties in different experimental models (Table 1).

**Table 1:** Anti-aging effect of LBP

**Animal studies**								
Authors	Year	Animal model	Dosage of LBP		Treatment approach		Key findings	Remarks
Tang R, et al. [29]	2019	*Drosophila melanogaster*	20 mg LBP-2, 40 mg LBP-1, and 200 mg LBP, in 100 g of the basal medium.		Feeding		LBP, LBP-1, and LBP-2 all significantly extended the average life span of *drosophila melanogaster*. Besides, LBP can increase the antioxidant activity of both 7 days and 21 days old drosophila. It increases the level of SOD and CAT and decreases the level of MDA. Among them, LBP-2 is the most effective one.	
Zhang Z, et al. [114]	2019	*C. elegans*	0, 200, 300, 400, 500 μg/mL		Exposed to different concentrations of LBP		LBP can extend the life of c. elegans by regulating sir2.1, daf-12 and daf-16, and the optimal concentration is at 300 μg/ml. LBP can increase the expression levels of SOD and CAT in c. elegans.	
Yang L, et al. [115]	2019	Cryopreserved mice embryo	50, 100, 200, 400, 800, and 1600 μg/ml		Exposed to different concentrations of LBP		The concentration of LBP at 1600 μg/ml is too high to delay the embryo growing. Choosing 200 μg/ml as the treatment dosage. LBP inhibit the mitochondria clustering, reduce the level of ROS and increase the mtDNA copy number, expression of sirtuin-1 (SIRT1) and AMPK. Besides, it can enhance the expression of GPX4, SOD1 and Bcl-2.	
Zhou J, et al. [112]	2016	X-ray induced mice	50, 100, and 200 mg/kg		Intraperitoneal injection		The rate of apoptosis in BMNC of mice, the G0/G1 ratio and the MDA levels are continuously decreased after LBP treatment, whereas SOD activity is increased.	Compared with the normal saline group, there was no significant difference with 50 mg/kg LBP group, while there was a significant difference in the other LBP groups.
Zhao R, et al. [40]	2015	Sub-health mice	10, and 20 mg/kg		Gastric infusion		LBP can increase the level of SOD and GSH-px and decrease that of MDA in skeletal muscle tissue. After treatment, mitochondrial membrane potential and the mitochondrial Ca^2+^ were increased. A higher concentration of LBP works better.	
Xia G, et al. [116]	2014	Zebrafish embryo	1.0, 2.0, 3.0 and 4.0 mg/ml		Continuously exposed to different concentrations of LBP		LBP showed significant resistance to replicating senescence at a concentration of 3.0 mg/ml. It can inhibit the expression level of p53, p21, and Bax, increase the expression level of Mdm2 and TERT, inhibit the apoptosis and death of zebrafish cells in early development, and alleviate the ageing of zebrafish.	
Yi R, et al. [117]	2013	D-gal ageing mouse	LBP water solution (10, 20, 40 ml/100 g·d) for continuous 30 days (unspecified)		Gastric infusion		LBP can increase SOD, CAT and GSH-px levels in the blood and reduce MDA level. LBP can improve skin SOD activity, reduce skin MDA content, and increase Hyp content.	
Xia G, et al. [118]	2012	Zebrafish embryos	0.125 mg/ml		Continuously exposed to LBP for 3 days.		LBP plays delaying senescence and prolonging the lifespan roles in zebrafish embryos model according to increase the expression of Mdm2 and TERT gene, meanwhile decrease the expression of Bax, p21, and p53 gene.	
Shan X, et al. [119]	2011	SD Rats	100, 200 and 400 mg/kg		Oral treatment		After LBP treatment, the average endurance time of the rats was significantly prolonged, which was also dose dependent. Besides, LBP decreases the level of MDA, meanwhile increases the level of SOD and GPx in a dose-dependent manner.	
Liang B, et al. [120]	2011	Aged rats	200 and 400 mg/kg		Oral treatment		LBP increase the level of SOD, CAT, and GSH-Px and decreases the level of MDA in a dose-dependent manner.	
Li XM, et al. [121]	2007	Aged mice	200, 350 and 500 mg/kg		Gastric infusion		LBP can reduce endogenous lipid peroxidation in ageing mice, enhance antioxidant enzyme activity, and restore immune function. It increased the expression of SOD, CAT, GSH-Px and the total antioxidant capacity in the tested organs and decreased the expression of MDA and LPF.	The antioxidant activity of LBP is like that of vitamin C, however, at the same dosage, LBP is much better than vitamin C. There is a synergy between LBP and vitamin C
Li B, et al. [122]	2006	Acetic lead (Pb (Ac)2) induced mice	10, 15 and 20 mg/kg		Gastric infusion		LBP could inhibit the micronucleus rates of the mice’s marrow cells in a dose-dependent manner.	A micronucleus is a form of chromosomal aberration, and the study indicates that LBP can reduce DNA damage.
Hong-Bin D, et al. [123]	2003	D-gal ageing mouse	100 mg/kg		Unspecified		Both LBP and ABP can reduce the level of AGE, IL-2 and increase the spontaneous motor activity, memory ability, and learning ability. Besides, LBP and ABP improve lymphocyte proliferation and SOD activity.	
**Clinical studies**								
Authors	Year	Study Design	Sample size	Population	Groups	Dosage of LBP	key findings	Limitations
Amagase H, et al. [18]	2009	A double-blind, placebo controlled RCT	50	Chinses	GoChi group: 60 mL of gouqi juice twice daily (total, 120 mL/d) vs placebo group	1632 mg/daily serving (120 mL) of LBP	Compared with the placebo group, serum SOD and GSH-Px was significantly higher, and MDA had decreased in the GoChi group. Relative to the preintervention levels, GoChi group had a significant change in the SOD, GSH-Px, and MDA levels. Nevertheless, in the placebo group, these differences were not statistically significant.	
Li B, et al. [122]	2006	Prospective case series	22	Chinses	Before-after study in the same patient	100 mg of LBP twice a day (total, 200 mg/d).	After taking LBP, the speed of DNA repair was significantly improved, compared with that before taking LBP; the difference was very significant.	The experimental population were all working in a rubber factory, which may lead to a higher risk of DNA mutations. However, the shortcoming is a small sample size and short administration period.

#### In vitro studies

According to literature, LBP could scavenge free radicals e.g., ABTS free radical, DPPH free radical, superoxide anion and hydroxyl radical. This will reduce oxidative stress and plays an important role in delaying ageing [18, 27, 124]. Another study found that increasing concentrations of LBP resulted in a higher free radical scavenging rate. However, when the dose was around 100 to 250 μg/ml, the scavenging rate gradually reached a plateau [124].

LBP has demonstrated antioxidizing and anti-apoptotic effects in vitro [28, 38, 39, 125]. By reducing oxidative stress and other harmful factors related to ageing, LBP can reduce DNA fracture damage, strengthen cell activity, and achieve anti-ageing effects on cells. LBP was able to upregulate the nuclear factor E2-related factor 2 (Nrf2) [38, 39, 125] and induce translocation of cytoplasmic Nrf2 to the nucleus to bind to the antioxidant response element (ARE) [38]. Moreover, LBP could down-regulate the expression of the transcription inhibitor Bach1, reducing the competition with Nrf2 for binding to ARE. In return, it boosted the expressions of antioxidant enzyme genes [28, 38, 125].

The anti-ageing effect of LBP is dose dependent. The effective dose of LBP in vitro was around 200 μg/ml. At this dosage LBP inhibits mitochondria clustering, reduces the level of ROS, and increases the mtDNA copy number and the expression of sirtuin-1 (SIRT1), AMPK, GPX4, SOD1 and Bcl-2 [115]. However, at a concentration of 1600 μg/ml LBP delayed the growth of murine two-cell embryos [115]. It is suggested that the dosage was critical for the anti-ageing effects of LBP.

#### Animal studies

The anti-ageing effect of LBP in vivo has been widely discussed (Table 1), with mice as most commonly used animal model.

In ageing mouse models, studies found that LBP reduced the level of advanced glycation end products (AGE) and IL-2. It also enhanced the memory and the learning ability of ageing mice [123]. Moreover, LBP could also promote the proliferation of lymphocytes and the activity of SOD [123]. It was suggested that LBP was involved in activating antioxidant enzyme activity in cells [123]. The contents of ROS and MDA were found to be decreased while the activities of antioxidant enzymes like SOD, CAT and GSH-Px were found to be increased in a dose-dependent manner in the aged mice treated with LBP [120, 121]. This strongly suggested that LBP can regulate the level of oxidation products and the antioxidant enzyme activity. Later, some studies revealed that LBP might delay oxidative stress induced animal ageing [117, 119, 126]. LBP might reduce DNA damage and inhibit the micronucleus rates in the mice’s marrow cells in a dose-dependent manner [122]. Moreover, LBP could reduce the G0/G1 ratio of bone marrow mononuclear cells (BMNC) and inhibit apoptosis of BMNC in mice [126].

LBP could delay senescence as well as prolong the lifespan of zebrafish embryos [116, 118]. It inhibited apoptosis of zebrafish embryos during early development and alleviated the ageing of zebrafish by blocking p53 signalling pathway [116, 118]. It increased the expression of murine double minute 2 (Mdm2) while decreasing the expression of Bax, p21, and p53 gene [116, 118]. On the other side, LBP has been observed to enhance the expression of the telomerase reverse transcriptase (TERT) which regulates and catalyses telomerase activity to maintain and extend the telomere structure of the chromosome in zebrafish embryos [116, 118]. This strongly demonstrates that LBP can alleviate telomeres shortening, a widely knowledgeable key factor associated with the ageing phenomenon, to delay cellular senescence.

Although mouse and zebrafish models are prevalent in LBP research, the effects of LBP have recently been investigated in other in vivo models, such as *Drosophila melanogaster* [29] and *C. elegans* [114]. All the experiments confirm that LBP has the ability to extend the average lifespan. Apart from increasing the expression level of antioxidant enzymes and reducing the content of MDA, LBP has also been found to regulate the lifespan through sir2.1, daf-12, and daf-16 [114].

#### Clinical studies

In line with the in vitro and in vivo studies, clinical studies also demonstrated that LBP as an antioxidant reduced DNA damage effects. The DNA repair rate was significantly improved for those who consumed LBP [122]. Relative to the placebo group, serum SOD and GSH-Px were significantly higher, while MDA decreased in the LBP group [18]. It reveals that LBP has the same effect on humans as on animals. This provides strong evidence for future studies on the anti-ageing effect of LBP in humans.

**Fig. 3:**
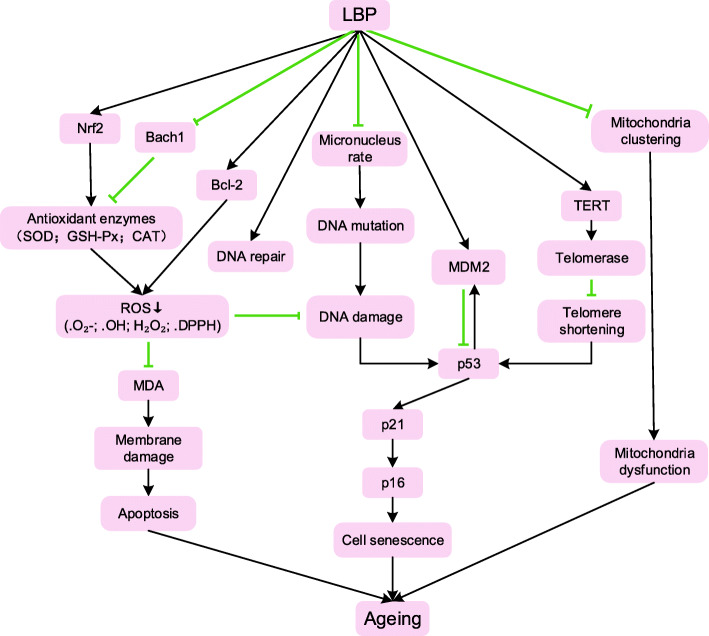
Schematic illustration of anti-ageing effects of LBP in the literature. LBP (*Lycium barbarum* polysaccharide); Nrf2 (nuclear factor E2-related factor 2); Bach1 (BTB domain and CNC homolog 1); Bcl-2 (B-cell lymphoma 2); GSH-Px (glutathione peroxidase); SOD (superoxide dismutase); CAT (catalase); ROS (reactive oxygen species); DPPH (2,2-Diphenyl-1-picrylhydrazyl); MDA (malondialdehyde); DNA (deoxyribonucleic acid); MDM2 (murine double minute 2); TERT (telomerase reverse transcriptase)

Collectively, LPB, a potent antioxidant, possesses an anti-ageing potential through regulating the level of oxidation products, the antioxidant enzyme activity, DNA damage, mitochondrial function, and cellular senescence (Fig. 3).

### Potential anti-OA effect of LBP

In recent years, LBP proved to have a beneficial effect on OA through its anti-inflammatory effects (Table 2). It was reported that LBP significantly reduced the levels of IL-1 β, TNF-α, iNOS and NF-κB p65 in the supernatant of OA chondrocytes [56]. LBP could upregulate miR-124 to reverse IL-1β induced upregulation of Cox-2 and inflammatory cytokines such as IL-6 and IL-8 [54]. This supported the notion that LBP could inhibit NF- κB signalling pathway and IL-1β evoked inflammatory injury in vitro. When LBP was added to palmitate-induced MC3T3-E1 osteoblast cells, the apoptosis was significantly reduced in a dose-dependent manner [55]. It was revealed that LBP could decrease the expression of Caspase-3, Caspase-9, Caspase-12, GRP78 and CHOP [55]. The above results showed that LBP exerted anti-inflammatory effects by inhibiting the JNK and NF-κB signalling pathways. Moreover, LBP could reduce paw thickness and protect bone integrity [57].

**Table 2:** Potential anti-OA effects of LBP

**Cellular studies**						
Authors	Year	Model	Dosage of LBP	Measured approach	Key findings	Limitations
Ni H, et al. [54]	2019	IL-1β evoked inflammatory injury in ATDC5 cell	300lg/mL LBP	The expression of TNF-α, IL-6, IL-8, Cox-2 and miR-124	LBP could decrease the expression of TNF-α, IL-6 and IL-8, and inhibit the expression of Cox-2, which evoked by IL-1β. LBP could increase the concentration of miR-124, then inhibited the activation of JNK and NF-κB pathways.	
Jing L, et al. [55]	2018	Palmitate-induced apoptosis in MC3T3-E1 osteoblasts cells	0, 50, 100, 200, 400 and 800 μg/ml	Cell viability, apoptotic rate and the expression of apoptosis-related genes.	LBP could inhibit palmitate-induced apoptosis which is in a dose-dependent manner, and the activation of the JNK pathway. LBP could decrease the expression of Caspase-3, Caspase-9, Caspase-12, GRP78 and CHOP.	
Cai ST, et al. [56]	2018	Human OA chondrocytes	0, 100, 200, 400 and 800 μg/mL	Expression of inflammatory cytokines of OA chondrocytes	LBP could inhibit the proliferation of OA chondrocytes in a concentration-dependent manner. 400 μg/mL LBP significantly reduced the levels of IL-1 β, TNF-α, iNOS and NF-κBp65 in the supernatant of OA chondrocytes and increased TGF-β expression. LBP could inhibit NF- κ B signal pathway and the inflammatory response of OA chondrocytes cultured in vitro.	
**Animal studies**						
Authors	Year	Animal model	Dosage of LBP	Treatment approach	Key findings	Limitations
Liu Y, et al. [57]	2015	collagen type II-induced arthritis mouse model	25, 50 and 100 mg/kg	Intraperitoneal injection for continuous10 days, once daily. The expression of TNF-α, IL-6, IL-17, MMP-1 and MMP-3. To observe the bone morphology and measure the paw diameter.	LBP could reduce the paw thickness, protect the bone integrity, and decrease the expression of TNF-α, IL-6 and IL-17, which are all in a dose-dependent manner. LBP could reduce the expression of MMP-1 and MMP-3 but not in a dose-dependent manner.	

To date, LBP treatment has rarely been applied to OA models in vivo. In the murine collagen type II-induced arthritis model, it was found that LBP decreased the expression of inflammatory mediators TNF-α, IL-6 and IL-17 in a dose-dependent manner. Additionally, it could reduce the expression of matrix-degrading enzymes MMP-1 and MMP-3 [57]. Therefore, LBP can protect the skeletal integrity of mice by reducing inflammation, significantly alleviating collagen type II-induced arthritis in mice.

**Fig. 4:**
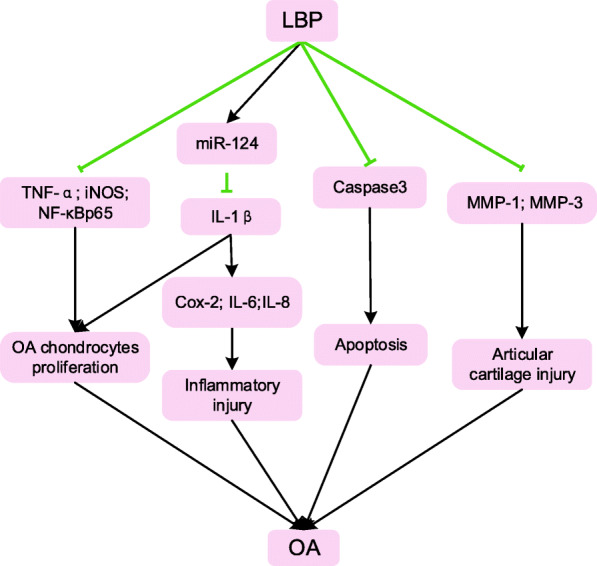
Schematic illustration of potential anti-OA effects of LBP in the literature. LBP (*Lycium barbarum* polysaccharide); TNF-α (tumour necrosis factor-α); iNOS (inducible nitric oxide synthase); NF-κBp65 (nuclear factor kappa-B); OA (osteoarthritis); miR-124 (microRNA-124); IL-1β (interleukin-1β); Cox-2 (cyclooxygenase-2); IL-6 (interleukin-6); IL-8 (interleukin-8); MMP-1 (matrix metalloproteinase-1); MMP-3 (matrix metalloproteinase-3)

In brief, LBP potentially exerts an anti-inflammatory effect against age-related OA, which is achieved by inhibiting the JNK and NF-κB signalling pathways as well as by down-regulating inflammatory factors (Fig. 4). The LBP treatment was found to be effective in OA.

## Discussion

Osteoarthritis is a degenerative disease accompanied by chronic pain which seriously affects patients’ life quality. There are several factors that promote the onset of OA such as joint injury, ageing and obesity. According to the previous research, cellular apoptosis and abnormal autophagy are also factors that mainly respond to OA occurrence [127–129]. In addition, many findings have also studied the key role of metabolism in OA progress [130–132]. However, the whole pathogenesis of OA development is still largely unknown at this moment. Current treatment of OA relies on pain killers for pain relief, there are no disease-modifying drugs up till today. This review mainly focused on the pathogenesis of OA caused by ageing, including mitochondrial dysfunction, cellular senescence, and inflammaging [12, 101], trying to find a novel trend for OA therapy.

Recently, researchers tried to specifically block ageing-related hallmarks to prevent joint damage, for example, by removing senescent chondrocytes [94]. This approach showed promising results and might be the new trend for OA therapy. Based on this approach, LBP, an anti-ageing component, could be used to mitigate OA progression. With its anti-oxidizing and anti-inflammatory ability, LBP can lower oxidative stress and inflammation both locally and systemically which can improve OA progression [57]. The beneficial effect of LBP on OA could potentially be achieved through a synergistic interaction of multiple pathways. However, more research is needed to further deepen the knowledge on the senolytic pathways of LBP in OA joints.

To date, LBP has been studied extensively and there is a growing body of evidence indicating that LBP exerted anti-ageing properties. It has been largely studied in the treatment of glaucoma, macular degradation, and other age-related disorders in liver, kidney and heart [17], whereas the role of LBP on OA remains poorly understood. Current understanding of LBP in OA treatment is limited to its anti-inflammatory effects [57]. The effects of LBP on other ageing hallmarks in OA have seldomly been explored. In the existing studies, the underlying mechanism has not been explored, nor have appropriate experimental models been selected. Whether LBP can also be used as a potential treatment drug for OA is still unknown and worth further research.

Here we summarise the characteristics of LBP and OA in terms of ageing, propose a hypothesis of how LBP can improve OA and postulate the Chinese herb, i.e., LBP, as a novel disease-modifying drug for OA therapy.

**Fig. 5:**
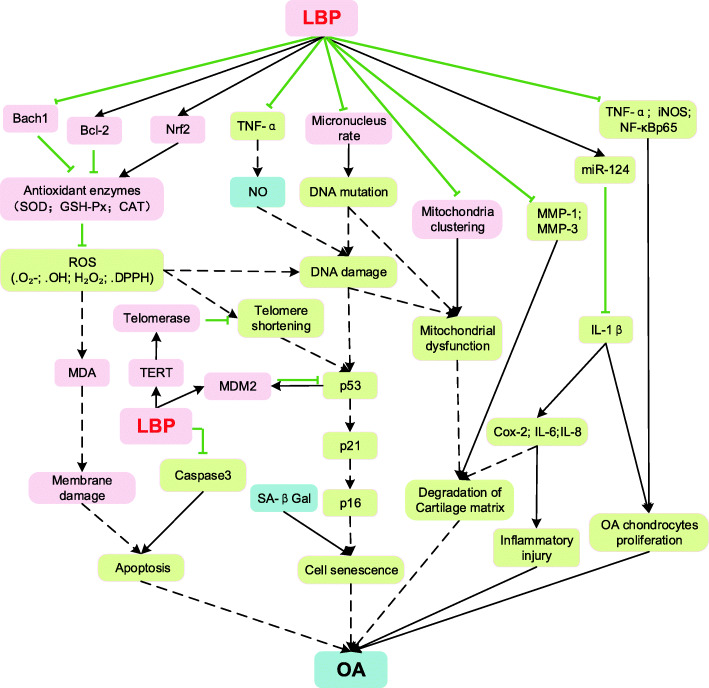
Proposed preventive and therapeutic mechanism of LBP in OA Factors related to anti-ageing effect of LBP are shown in pink; factors that are related to OA but not yet proved to be related to LBP is shown in blue; largely overlapped working mechanism of LBP with pathomechanims of OA (in yellow). It points to a direction that LBP is an emerging disease-modifying drug candidate for OA therapy. LBP (*Lycium barbarum* polysaccharide); Bach1 (BTB domain and CNC homolog 1); Bcl-2 (B-cell lymphoma 2); GSH-Px (glutathione peroxidase); SOD (superoxide dismutase); CAT (catalase); ROS (reactive oxygen species); DPPH (2,2-Diphenyl-1-picrylhydrazyl); MDA (malondialdehyde); Nrf2 (nuclear factor E2-related factor 2); TNF-α (tumor necrosis factor-α); NO (nitric oxide); DNA (deoxyribonucleic acid); MDM2 (murine double minute 2); TERT (telomerase reverse transcriptase); SA-βgal (Senescence-associated beta-galactosidase); MMP-1 (matrix metalloproteinase-1); MMP-3 (matrix metalloproteinase-3); miR-124 (microRNA-124); IL-1β (interleukin-1β); Cox-2 (cyclooxygenase-2); IL-6 (interleukin-6); IL-8 (interleukin-8); iNOS (inducible nitric oxide synthase); NF-κBp65 (nuclear factor kappa-B); OA (osteoarthritis)

Collectively, all listed works support the hypothesis that LBP could modify age-related OA and after careful study of the available research the potential underlying mechanism could be identified (Fig. 5). LBP can upregulate Nrf2, induce translocation of Nrf2 from cytoplasm to nucleus, and bind with ARE to promote the expression of antioxidant enzyme genes. Due to the presence of a large number of antioxidant enzymes (SOD, GSH-Px, CAT), ROS are eliminated. Consequently, LBP can alleviate the condition of OA by inhibiting the consequences related to oxidative damage i.e., DNA damage, telomere attrition, mitochondrial dysfunction, cellular senescence and inflammaging. First, LBP may block the p53-induced cellular senescence pathway and inhibit the development of OA by improving the DNA repair levels and protecting telomere integrity. Second, LBP may help to maintain the integrity of articular cartilage by protecting the function of mitochondria and reducing the degradation of the cartilage matrix. Third, LBP may inhibit caspase-3 expression, which is involved in chondrocyte apoptosis, to reverse the OA phenomenon. Fourth, LBP can trigger inflammatory protection mechanisms that reduce inflammatory cytokine levels, thereby relieving the symptoms of OA.

Given the above results, LBP theoretically has a positive effect on OA. Therefore, in the future, the focus will be on how to construct suitable experimental models to study the effect of LBP on OA.

## Conclusions

In conclusion, this review clarified the potential interaction between the basic pathological mechanism of OA and the anti-ageing effect of LBP. LBP showed a potential advantage in the treatment of OA due to its impact on multiple signalling pathways leading to lowering oxidative stress, restoring mitochondrial function, mitigating DNA damage, and preventing cellular senescence. Hence, we have a strong reason to believe that LBP, not only has well-documented antioxidant and anti-ageing effects but also may exert beneficial effects on OA treatment.

## Data Availability

Not applicable.

## Abbreviations

ATP: Adenosine triphosphate
ACSCs: Articular cartilage stem cells
Ad-hMSCs: Adipose tissue-derived human MSCs
AGEs: Advanced glycation end products
AMPK: AMP-activated protein kinase
AOPPs: Advanced oxidation production products
ARE: Antioxidant response element
Bach1: BTB domain and CNC homolog 1
Bcl-2: B-cell lymphoma 2
bmMSC: Bone marrow-derived mesenchymal stem cells
BMNC: Bone marrow mononuclear cells
CAT: Catalase
CHOP: C/−EBP homologous protein
Cox-2: Cyclooxygenase-2
DAMPs: Damage-associated molecular patterns
DNA: Deoxyribonucleic acid
DPPH: 2,2-Diphenyl-1-picrylhydrazyl
GPX4: Glutathione Peroxidase 4
GRP78: 78-kDa glucose-regulated protein
GSH-Px: Glutathione peroxidase
HMGB1: High-mobility group box 1 protein
IGF-1: Insulin-like growth factor-1
IL: Interleukin
IL-1β: Interleukin-1β
iNOS: Inducible nitric oxide synthase
JNK: Jun amino-terminal kinases
LB: *Lycium barbarum*
LBP: *Lycium barbarum* polysaccharide
MAPK: Mitogen-activated protein kinases
MDA: Malondialdehyde
Mdm2: Murine double minute 2
miR-124: MicroRNA-124
MMP: Matrix metalloproteinase
MRC: Mitochondrial respiratory chain
MSCs: Mesenchymal stem cells
mtDNA: Mitochondrial DNA
NF-κB: nuclear factor kappa B
NO: Nitric oxide
Nrf2: Nuclear factor E2-related factor 2
OA: Osteoarthritis
ROS: Reactive oxygen species
SASP: Senescence-associated secretory phenotype
SA-βGal: Senescence-associated beta-galactosidase
SIRT1: Sirtuin-1
SnCs: Senescent cells
SOD: Superoxide dismutase
TERT: Telomerase reverse transcriptase
TGF-β: Transforming growth factor-β
TNF-α: Tumour necrosis factor-α
